# How do people with long COVID utilize COVID-19 vaccination and rehabilitation services and what are their experiences with these services? results of a qualitative study with 48 participants from Germany

**DOI:** 10.1186/s12889-024-18380-6

**Published:** 2024-03-28

**Authors:** Tim Schmachtenberg, Gloria Königs, Sascha Roder, Frank Müller, Christina Müllenmeister, Dominik Schröder, Iman El-Sayed

**Affiliations:** 1 Department of General Practice, University Medical Center Göttingen, Humboldtallee 38, 37073 Göttingen, Germany; 2 Department of Rheumatology and Immunology, Hannover Medical School, Carl-Neuberg-Str. 1, 30625 Hannover, Germany; 3 Department of Social Work, University of Applied Sciences and Arts Bielefeld, Interaktion 1, 33619 Bielefeld, Germany; 4 Department of Family Medicine, College of Human Medicine, Michigan State University, 15 Michigan St NE, Grand Rapids, 49503 East Lansing, MI USA

**Keywords:** Long COVID, SARS-CoV-2, COVID-19 vaccination, Rehabilitation, Germany, Qualitative study

## Abstract

**Background:**

Studies estimate that at least 7.5% of adults are affected by long-term symptoms such as fatigue or cognitive impairment after the acute phase of COVID-19. COVID-19 vaccination may reduce the risk of long COVID. Rehabilitation can have a positive impact on recovery. This study aims to present the experiences of people with long COVID with COVID-19 vaccination and rehabilitation. Such research is important because perceptions of these measures can impact healthcare utilization and health status.

**Methods:**

48 adults with long COVID participated in this qualitative study, 25 of them in one-on-one interviews and 23 in focus groups. Participants were recruited via calls for participation on the websites and social media channels of two university hospitals and with the help of respondents’ networks. The conversations were audio-recorded, transcribed, and analyzed using qualitative content analysis. Subsequently, the results were compared, interpreted, and discussed by scientific literature.

**Results:**

35 study participants reported that they had received a COVID-19 vaccination and 16 of them stated that they had utilized a rehabilitation service. These participants had varying experiences with COVID-19 vaccination and rehabilitation. Nine of them stated that they developed long COVID despite vaccination before COVID-19. Ten participants reported vaccine reactions, and two participants reported severe side effects. Two participants reported persistent deterioration of their long COVID symptoms after vaccination. This led to uncertainty about the safety, benefits, and handling of COVID-19 vaccination. However, most participants perceived the vaccine as effective regarding milder COVID-19 sequelae. Four participants felt their rehabilitation was helpful and four participants felt it was unhelpful. Two persons found the combination of inpatient rehabilitation and rehabilitation sport helpful.

**Conclusions:**

Several implications can be derived from this study: (1) researchers should explore the effects of COVID-19 vaccination on long COVID symptoms; (2) vaccination campaigns should be more responsive to the perspectives of people with long COVID on vaccination; (3) care planners should build rehabilitation facilities specialized in long COVID; (4) rehabilitation providers should train their professionals regarding long COVID and develop rehabilitation programs tailored to different clinical pictures.

**Trial registration:**

German register for clinical trials DRKS00026007, 09 September 2021.

**Supplementary Information:**

The online version contains supplementary material available at 10.1186/s12889-024-18380-6.

## Background

More than 676 million people worldwide have been infected with SARS-CoV-2 (severe acute respiratory syndrome coronavirus type 2) [1]. In Germany, the number of COVID-19 cases reported by public health authorities since the start of the pandemic is approximately 38 million (as of 10 March 2023) [2]. Current estimates indicate that at least 7.5% of adults have persistent symptoms following SARS-CoV-2 infection [3]. For this phenomenon, the literature uses terms such as *post-* and *long COVID-19*. In this paper, the term “long COVID” proposed by the German Federal Institute of Public Health Robert Koch Institute (RKI) is used [4]. Following the guidelines of the UK National Institute for Health and Care Excellence (NICE) [5], the RKI defines long COVID as health symptoms that persist for more than four weeks beyond the acute phase of illness of SARS-CoV-2 infection or are new onset after that period [4]. The most common symptoms of long COVID are fatigue, concentration problems, shortness of breath, muscle pain, and psychological problems [6–12]. Immunological processes causing persistent inflammation are assumed to be the cause of the disease [13]. Currently, no treatments targeting the cause of the condition have been approved [7]. Research suggests that adapted rehabilitation interventions have a positive impact on the recovery process of people with long COVID [14–16]. Furthermore, studies show that first rehabilitation programs developed for long COVID patients [16] and certain existing programs adapted to the needs of those affected (e.g. pulmonary or physical exercise-based rehabilitation) can lead to an improvement in individual symptoms (e.g. dyspnea or depression) [17–19]. However, the current evidence on the efficacy of rehabilitation programs for long COVID complaints and the patients’ perception of such services is still fragmentary [20, 21]. Nevertheless, affected patients receive rehabilitative measures. This study aims to address the knowledge gap regarding this patient population’s rehabilitative experiences.

A preventive measure to protect the population from the health consequences of COVID-19, which has been supported by various public campaigns at the international and national levels, is the COVID-19 vaccination [22, 23]. In Germany, more than 76% of the population is now immunized (as of 10 March 2023) [24]. Studies suggest that vaccination against COVID-19 may reduce the risk of long COVID [25–27]. COVID-19 vaccinations can be associated with vaccine reactions, side effects, or vaccine complications, although these are usually short-lasting mild symptoms such as erythema or swelling at the injection site [28, 29]. The extent to which people who already have long COVID or have developed long COVID despite COVID-19 vaccination perceive this measure as helpful for their health has not yet been adequately addressed. This question is of particular importance to identify and address possible developing issues for individual and public health, such as vaccination hesitancy resulting from insecurities, within this population at an early stage.

The aim of this qualitative study is to show to what extent people with long COVID utilize COVID-19 vaccination and outpatient or inpatient rehabilitation services, what their experiences are with these services and how they perceive the impact on their health. COVID-19 vaccination and rehabilitation are considered together in this study, as these measures are among the few effective interventions regarding the prevention or alleviation of long-term symptoms after SARS-CoV-2 infection and are subsequently associated with high expectations for many people with long COVID. Negative experiences and uncertainties regarding these measures can negatively impact the utilization of the services and the own health status of affected individuals. The work complements the scientific discourse with the perspective of people from a population group particularly affected by the health consequences of the COVID-19 pandemic on the central preventive measure of the German healthcare system regarding the health-related pandemic consequences [22]. Moreover, the study supplements the existing literature by describing the perception of rehabilitation measures by people who have an increased need to reduce or eliminate illness-related impairments [30]. Experiences with such key public health services during a situation of high disease burden and high support needs may influence the attitudes and behaviors of affected individuals regarding formal health services and care providers. To increase trust in the healthcare system and minimize public health consequences, it may be important to collect such experiences and consider the concerns of people with long COVID in future measures such as vaccination campaigns or the development of rehabilitation programs.

## Methods

This is a qualitative study with 25 participants from one-on-one interviews and 23 participants from four focus groups. The study is part of the multicenter research project “DEFEnse Against COVID-19 STudy - Looking forward” (DEFEAT Corona) [31]. Central topics of the guided interviews and moderated focus groups were their health situation, the impact of the disease on everyday life, and medical care. Inclusion criteria of the study were having long COVID according to the RKI definition (symptoms persisting beyond the acute phase of SARS-CoV-2 infection of four weeks and not explained by any other diagnosis [4]) and a minimum age of 18 years. Furthermore, consent to participate in a videoconference or in-person interview was required for the one-on-one interviews, and consent to participate in a face-to-face discussion round was required for the focus groups. Individuals who did not have persistent COVID-19 symptoms or whose SARS-CoV-2 infection was less than 4 weeks ago, minors, individuals who did not consent to participate in the interviews or focus groups, and individuals whose cognitive or physical impairment was too severe to conduct a one-hour interview or group discussion were not included. The publication is following the Consolidated Criteria for Reporting Qualitative Research (COREQ) [32].

### Recruitment

Two different cohorts were recruited for this study. Recruitment of participants for both samples was carried out (a) via a call for participation on the websites of the Department of General Practice of the University Medical Center Göttingen (UMG) and the Hannover Medical School (MHH), (b) via posts on the social media channels (Instagram and Facebook) of the involved universities, and (c) with the help of respondents who suggested other people for participation (snowball system). To recruit participants for the one-on-one interviews, additional (d) information flyers about the study were distributed in general practices in Lower Saxony and at the Public Health Authority of the City of Göttingen, and (e) invitation letters (*n* = 130) were sent to individuals who had already participated in an earlier subproject [33] of DEFEAT Corona. Recruitment activities for the one-on-one interviews occurred in January and February 2022 and for the focus groups in May and June 2022. Interested individuals were contacted by phone or email prior to the interviews to verify inclusion and exclusion criteria and to provide study education. Prior to the interviews, recruited individuals provided written informed consent to participate in the interviews or focus groups and for data publication. The sample size was based on existing literature on good qualitative research practice, which recommends conducting at least twelve interviews [34–36] or three to five focus groups of five to eight people each [37–40]. Most of the participants in the focus groups did not know each other before data collection. The participants received a compensation of 40 Euros (approx. 40 USD) for their participation.

### Development of interview and moderation guidelines

The guideline for the one-on-one interviews was developed based on a workshop with six researchers, which was held at the Department of General Practice on 29 October 2021. The moderation guideline for the focus groups, which was developed in a discursive process by three authors (TS, SR, and IES) on 10 June 2022, was based on methodological literature [37–39] and the interview results. The following guiding questions were derived from the guidelines for the interviews and focus groups: (1) To what extent do people with long COVID perceive COVID-19 vaccination as a useful tool regarding health promotion? (2) How does this perception affect the utilization of the vaccination service? (3) To what extent do people with long COVID experience a temporary or permanent change in their health status and symptoms following COVID-19 vaccination? (4) To what extent do they perceive inpatient and outpatient rehabilitation services as helpful for their health and their long COVID-related complaints? Besides 26 open-ended questions, the interview guideline also included a section of closed questions to collect sociodemographic information. To provide a stimulus for discussion, a recorded radio report was played at the beginning of the focus groups on the topic of long-term consequences of COVID-19 in everyday life and medical support for long COVID [41]. Additionally, the researchers asked several open-ended questions and included a quote from the one-on-one interviews. Both guidelines are available as supplementary material. To obtain authentic insights into the current life situation of the participants, the researchers did not provide them with the guidelines in advance of the interview and focus group discussions.

### Data collection

The one-on-one interviews were conducted between January and May 2022, and the focus groups on 22 June and 6 July 2022. Twenty-one of the 25 one-on-one interviews were held using Zoom video conferencing software (Zoom Video Communications, Inc., San Jose, CA, USA). This setting was chosen to include individuals who are affected by mobility limitations due to their health conditions. Respondents participated in the videoconference from their homes. Four interviews and all focus groups were conducted at the Department of General Practice in Göttingen and the Hannover Medical School. In this way, interaction qualities that may be limited in a digital setting were considered. These include a deeper situational understanding and non-verbal communication [42]. During the focus groups and most of the interviews, no persons other than the participants and the researchers were present at the location where the data were collected. In several Zoom interviews, an artist was present as a silent observer, who supported the study team with a graphic interpretation of the statements of the participants [43]. The one-on-one interviews were conducted by one researcher (SR) and the focus groups by three researchers (TS, SR, and IES). In the focus groups, the researchers had alternating roles, with each person moderating, co-moderating, and taking minutes at least once. The online interviews were digitally recorded and the face-to-face interviews were recorded using an audio recording device. At the beginning of the focus groups, some sociodemographic data were collected. Furthermore, the researchers noted content-related conspicuities and the sequence of speakers during the conversations.

### Data evaluation

The data from both samples were analyzed on the same methodological basis and with the help of identical analysis steps. First, the content-semantic transcription of the audio recordings was carried out [44, 45]. Then, a team of three researchers with a social science (TS and SR) or social work (IES) background and three study assistants (including GK) conducted a qualitative content analysis [46, 47]. For quality assurance, a consensual approach was used in which the individual analysis steps were carried out simultaneously by several members of the study team and the results were subsequently discussed. After the initiating text work, two coding processes occurred. Thereby, the study team developed two separate category systems for the one-on-one interviews and the focus groups using a combination of deductive and inductive categorization. The main categories were deductively derived from the interview or moderation guidelines. During the initial coding process, top categories and subcategories were inductively derived from the material and axially linked. After the finalization of the category systems and the discursive development of codebooks with category definitions, coding rules, and anchor examples, the entire material was coded again. For both coding processes, the study team used MAXQDA software version 20.0.8 (VERBI Software GmbH, Berlin, Germany). Table 1 shows the excerpts from the category systems relevant to this study, each consisting of three levels. Both the five main categories from the one-on-one interview data and the eleven main categories regarding the focus groups are divided into 33 top and subcategories. This study includes the categories of therapeutic measures/rehabilitation experiences, COVID-19 vaccination, and side effects of COVID-19 vaccination. After coding, the original statements of the study participants were paraphrased and generalized. Finally, thematically relevant quotes and generalizations from all one-on-one interviews and focus groups were compiled into tables, compared, interpreted, and then discussed by scientific literature.

**Table 1:** Excerpts from the category systems

Sample	Main categories	Top categories	Subcategories
Interviews	1. Health situation		
		1.1 Current state	
		1.2 COVID-19 infection	
			1.2.1 Assessment of personal risk
			1.2.2 Consequences of COVID-19 infection
			1.2.3 Improvement of the complaints
			1.2.4 Therapeutic measures
		1.3 COVID-19 vaccination	
			1.3.1 Immunization education
			1.3.2 Side effects of COVID-19 vaccination
		1.4 Healthcare orientation	
		1.5 Health behavior	
			1.5.1 Strategy development regarding health restrictions
Focus groups	2. Orientation in the healthcare system		
		2.1 Criticism of the healthcare system	
			2.1.1 Assessment of medical ability to act
		2.2 Positive experiences with medical care	
		2.3 Negative experiences with medical care	
		2.4 Diagnostic measures	
		2.5 Therapeutic measures	
			2.5.1 Rehabilitation experiences
		2.6 COVID-19 vaccination	
			2.6.1 Side effects of COVID-19 vaccination

### Healthcare system information

German law requires residents to have health insurance. While some groups of people, such as civil servants or the self-employed, can be insured privately, the vast majority of residents are compulsorily insured in the statutory health insurance system. They are entitled to free-of-charge treatment [48]. COVID-19 vaccinations are free of co-payment for all residents. The cost of the vaccine is covered by the federal government, and the cost of the vaccination centers was shared between the federal states and statutory and private health insurance [49]. Outpatient and inpatient rehabilitation measures, such as psychosomatic, neurological, or cardiological rehabilitation and rehabilitation for post- or long COVID, are paid for by pension funds. For this purpose, applications must be submitted and approved [50].

## Results

The main statements of the participants presented in this chapter are supported by original quotes from the interviews. While a few short quotes were integrated into the main text, some long quotes are listed in Table 3. Discussions were forward translated into English. The following notations at the end of quotes and in Tables 2 and 3 include: “F” stands for Focus Group, the “P” for Participants, and the “I” for Interviewees.

**Table 2:** Overview of sociodemographic characteristics of participants

Participant	Sex	Age	Occupation	Interview date (setting)
Interviews				
I1	Female	**28–37 years**	Physician	01/24/2022 (online)
I2	Male	**28–37 years**	Management Consultant	02/17/2022 (online)
I3	Female	**58–67 years**	Homemaker	02/18/2022 (online)
I4	Female	**38–47 years**	Clerk	02/21/2022 (online)
I5	Female	**38–47 years**	Referent	02/25/2022 (online)
I6	Female	**18–27 years**	College student	03/07/2022 (online)
I7	Male	**38–47 years**	Physical therapist	03/08/2022 (online)
I8	Male	**48–57 years**	Social pedagogue	03/18/2022 (online)
I9	Female	**28–37 years**	Climate Protection Manager	03/24/2022 (online)
I10	Female	**48–57 years**	Teacher	03/28/2022 (online)
I11	Male	**58–67 years**	Retiree	03/29/2022 (online)
I12	Female	**48–57 years**	Secretary	03/30/2022 (online)
I13	Male	**18–27 years**	College student	04/01/2022 (online)
I14	Female	**48–57 years**	Nurse	04/04/2022 (online)
I15	Female	**58–67 years**	Official	04/05/2022 (online)
I16	Female	**58–67 years**	Physician Assistant	04/07/2022 (online)
I17	Male	**28–37 years**	Service technician wind energy	04/12/2022 (online)
I18	Female	**48–57 years**	Teacher youth dental care	04/14/2022 (online)
I19	Female	**18–27 years**	Nurse	04/20/2022 (online)
I20	Female	**48–57 years**	Nurse	04/26/2022 (online)
I21	Female	**18–27 years**	Trainee therapy science	04/28/2022 (Göttingen)
I22	Female	**48–57 years**	Teacher	05/03/2022 (Göttingen)
I23	Male	**58–67 years**	Foreman construction	05/03/2022 (Göttingen)
I24	Female	**38–47 years**	Administrator	05/11/2022 (Göttingen)
I25	Female	**28–37 years**	Physician	05/14/2022 (online)
Focus groups				
F1, P1	Male	**48–57 years**	Banker	06/22/2022 (Göttingen)
F1, P2	Male	**48–57 years**	Hygiene manager	06/22/2022 (Göttingen)
F1, P3	Female	**48–57 years**	Nurse	06/22/2022 (Göttingen)
F1, P4	Female	**38–47 years**	Lab technician	06/22/2022 (Göttingen)
F1, P5	Male	**18–27 years**	College student	06/22/2022 (Göttingen)
F2, P1	Female	**No information**	Office clerk	06/22/2022 (Göttingen)
F2, P2	Female	**18–27 years**	Preschool teacher and student	06/22/2022 (Göttingen)
F2, P3	Female	**18–27 years**	Lab technician	06/22/2022 (Göttingen)
F2, P4	Female	**18–27 years**	Student	06/22/2022 (Göttingen)
F2, P5	Female	**58–67 years**	Board member of a company	06/22/2022 (Göttingen)
F3, P1	Female	**28–37 years**	Social worker	07/06/2022 (Hannover)
F3, P2	Female	**48–57 years**	Journalist	07/06/2022 (Hannover)
F3, P3	Male	**48–57 years**	Cook	07/06/2022 (Hannover)
F3, P4	Female	**48–57 years**	Medical assistant	07/06/2022 (Hannover)
F3, P5	Female	**38–47 years**	Employee in geriatric care	07/06/2022 (Hannover)
F3, P6	Male	**58–67 years**	Driver	07/06/2022 (Hannover)
F4, P1	Male	**48–57 years**	Engineer	07/06/2022 (Hannover)
F4, P2	Female	**28–37 years**	Beautician	07/06/2022 (Hannover)
F4, P3	Female	**48–57 years**	Teacher	07/06/2022 (Hannover)
F4, P4	Male	**48–57 years**	Police medical inspector	07/06/2022 (Hannover)
F4, P5	Female	**28–37 years**	Paramedic	07/06/2022 (Hannover)
F4, P6	Female	**28–37 years**	Preschool teacher	07/06/2022 (Hannover)
F4, P7	Female	**28–37 years**	Disability beneficiary (former office manager)	07/06/2022 (Hannover)

**Table 3:** Illustrative quotations for themes and statements derived from the interviews and focus groups

Theme	Statements	Example quotes
Perceptions of COVID-19 vaccination	One participant reported pronounced health symptoms and limitations in everyday life during the acute COVID-19 phase, despite having previously received a booster vaccination.	“I had for example […] a very, very severe course […] despite vaccination and despite a booster. […] I spent three weeks in bed, crawling on all fours to the toilet, which was really the highest of feelings. When I thought, ‘Okay, I’m starting to get better. I also tested negative’, I didn’t even make it down the stairs.” (F1, P4)
	Several participants stated that they had not been vaccinated at the time of infection due to the state-established vaccination order.	“When I had it last year [COVID-19], it […] was just starting with the vaccination campaign and I was not yet in the prioritization stage in terms of age that I could have been vaccinated.” (I4)
	Two participants reported that they did not make use of booster vaccinations for fear of negative effects on their long COVID complaints.	“I’ve gotten to the point where I don’t do it [get vaccinated a third time] because I’m afraid […] that I might not be able to think clearly if I get vaccinated again now.” (I3) “I was vaccinated twice and I would never get vaccinated again.” (F2, P2)
	The participants wished for scientific research on the question whether their symptoms could be a long-term consequence of the vaccination or whether there is an interaction between COVID-19 and COVID-19 vaccination that leads to long-term complaints.	“The question that’s developing for me right now from this is, can I attribute this [my symptoms] to the vaccination? Is this some late effect of the vaccination? I got boosted in December and […] I got COVID in mid-February. Or is that somehow the vaccine plus the disease, that that triggers something, some processes in the body, that that happens […]? […] Maybe we can find that out sometime.” (F2, P5)
	One participant described her perception that non-vaccinated individuals with SARS-CoV-2 infection from her social environment had better health than her with vaccination.	“I can only reflect what I experience from my immediate environment. And that is just, those who were not vaccinated, they are feeling better now despite infection than me with vaccination.” (F2, P2)
	Some participants were uncertain about the health benefits of COVID-19 vaccination for people with pre-existing long COVID. They expressed doubts about vaccination decisions al-ready made and about the future handling of this measure.	“Then I really asked myself ‘What did it [the COVID-19 vaccination] bring about, did it help at all now?’ So I really caught myself asking that, thinking to myself ‘If I hadn’t gotten vaccinated, would it have been better or worse?” (I13) “I am now also skeptical about this vaccination. Well, I could maybe also say possibly it would have been much worse if I hadn’t been vaccinated, but so a bit of mistrust is already growing.” (I11)
Vaccination reactions after COVID-19 vaccination	Three participants experienced comparable symptoms following COVID-19 vaccination to those during the acute phase of COVID-19.	“I have to say about that, afterward [after the vaccination] I felt as bad again as when I had COVID. So, I could breathe so badly again. […] That went on for a few days.” (I3)
	Two participants reported externally visible symptoms as a result of the vaccination.	“Because I had bruises all over my body after the first Biontech vaccination” (F4, P2). “Then [after vaccination] the swelling started […]. Which was pretty blatant with swelling of the genitals, feet, hands, face.” (I7)
	Two participants reported very poor health and heart problems following COVID-19 vaccination.	“I had heart problems after the booster vaccination, which meant I had also been in the hospital for heart muscle inflammation, at that time.” (I2) “I had heart palpitations in November 2021, after I got two vaccinations shortly after each other, […]. I was really really bad since the second vaccination. In August 2021 I got the second vaccination, then I just dragged myself to work, then I came home and couldn’t do anything. All of that reminded me of when I had COVID and was just dragging myself around. Then I had something like long COVID, I was just broken and didn’t want to hear anything. […] Then I had these lapses, they had then done an exercise ECG on me and that’s when they found the heart palpitations.” (I9)
	Two participants experienced a persistent deterioration in their health and long COVID symptoms after vaccination against COVID-19.	“I’ve also noticed through my booster vaccination […] how much worse my long COVID symptoms have become again.” (I5)
Rehabilitation experiences	Two participants perceived the combination of inpatient rehabilitation at the seaside and follow-up rehabilitation sports as helpful.	“I’ve been in rehab in the meantime, and it’s already done a lot of good for me. I was at the North Sea. […] After that, I also started doing rehab sports. I just notice that it does me good.” (I7)
	The participants discussed the lack of focus of rehabilitation clinics on long COVID and the lack of experience of professionals in treating patients with this condition as possible reasons for this.	“I also just recently had a rehab that unfortunately wasn’t very successful, so it wasn’t that great. There were quite a lot of things started, but not finished unfortunately.” (F4, P1) “Also relatively quickly sent to rehab […] to Borkum [an island in the North Sea]. It was still relatively early last year in summer [2021] […]. There was just a lot of lung training so that the lungs first get well again. But it has been found out that my lung has nothing at all […]. And the clinic was not specialized for long COVID. […] In retrospect, the sport I did was exactly the wrong thing. I did too much sport in that rehab.” (F3, P3)

### Description of cohorts

27 people responded to the call for participation in the one-on-one interviews. Finally, 25 interviews were evaluated. One person withdrew their consent to participate without noting reason and one participant canceled the interview due to health reasons. 39 individuals expressed interest in participating in the focus groups. Of the 35 individuals who met the inclusion criteria, 28 were randomly assigned to the sample. Three participants were not present on the day of the data collection without prior cancellation. Two further participants canceled at short notice, one of them due to health reasons. A total of 23 participants attended the focus groups.

The participants were between 19 and 67 years old (mean: 43.1 years), 34 were female (71%) and 14 were male (29%). The proportion of women (one-on-one interviews: 72%, focus groups: 70%) as well as the mean age of participants (44.5, 41.6) was slightly higher in the one-on-one interviews than in the focus groups. 41 participants (85%) were employed. Table 2 shows the selected socio-demographic characteristics of the participants (sorted by date of data collection).

The interviews had a mean duration of 38 min (min: 17 min, max: 57 min) and the focus groups lasted an average of 69 min (min: 60 min, max: 80 min). Content analysis identified themes in three categories: (1) perceptions of COVID-19 vaccination, (2) vaccination reactions after COVID-19 vaccination, and (3) rehabilitation experiences.

### Perception of COVID-19 vaccination

All 25 participants in the one-on-one interviews reported receiving vaccinations against COVID-19. A total of 23 stated receiving at least two vaccinations. In the focus groups, ten people highlighted that they had used this preventive measure. Eight of them pointed out being vaccinated against COVID-19 at least twice. The participant statements indicate a high willingness to be vaccinated and a high vaccination rate in this sample. None of the participants stated that they had rejected a vaccination offer.

There were differences among the participants regarding the sequence of COVID-19 vaccination and COVID-19, as well as their perception of the preventive measure. Nine participants reported that they became infected with SARS-CoV-2 and developed long COVID symptoms despite prior COVID-19 vaccination. However, two individuals were not fully immunized (only one vaccine shot) at the time of infection. One participant reported pronounced health symptoms and limitations in everyday life during the acute COVID-19 phase, despite having previously received a booster vaccination.

Six participants stated that they had not been vaccinated at the time of infection. Most of these participants cited the reason for this as due to the state-established vaccination order, stating that only over 80-year-olds and high-risk individuals were vaccinated first [51]. One person reported that her uncertainty about possible side effects led to a delayed vaccination decision. While the majority of twice-vaccinated individuals decided to get vaccinated a third time, two individuals decided not to do so based on the recommendation of their healthcare providers. *“I didn’t get the third one because I didn’t need it, they told me in rehabilitation” (I22).* Two participants reported that they did not make use of booster vaccinations for fear of negative effects on their long COVID complaints (intensification of existing complaints, occurrence of new cognitive symptoms). For them, further COVID-19 vaccination was not an option.

Two participants in the focus groups discussed a possible correlation between the COVID-19 vaccination and their health complaints. They asked themselves whether their symptoms could be a long-term consequence of the vaccination or whether there is an interaction between COVID-19 and COVID-19 vaccination that leads to long-term complaints. The participants wished for scientific research on this question. One participant described her perception that non-vaccinated individuals with SARS-CoV-2 infection from her social environment had better health than her with vaccination.

Overall, the number of participants who were convinced of the effectiveness of COVID-19 vaccination in terms of milder COVID-19 acute courses and long-term sequelae predominated. *“Probably also because of vaccination, the courses have been milder” (I2).* Five participants emphasized wanting to be vaccinated again. *“Would also take another [vaccination] at any time” (I19).*

However, several of the participants with a generally positive attitude toward COVID-19 vaccination also wondered whether this measure had a positive or negative impact on their health. Some participants were uncertain about the health benefits of COVID-19 vaccination for people with pre-existing long COVID. They expressed doubts about vaccination decisions already made and about the future handling of this measure.

### Vaccination reactions after COVID-19 vaccination

Overall, ten participants reported varying vaccination reactions in terms of type and severity after COVID-19 vaccination. Two of them stated that they had perceived these exclusively after the first vaccination. *“The [first vaccination] pretty much knocked me out for another three weeks. But the second vaccination I had no problems at all” (I20).* Three participants experienced comparable symptoms following COVID-19 vaccination to those during the acute phase of COVID-19 (fever, limb and muscle pain, breathing problems, severe fatigue). Two participants reported externally visible symptoms such as swelling or hematomas as a result of the vaccination. Two participants stated that they were in very poor health and had heart problems after the COVID-19 vaccination. One individual suffered from extrasystoles (heart palpitations) and other complaints that she perceived as comparable to the acute COVID-19 symptoms and her long COVID complaints. One interviewee was hospitalized with myocarditis (inflammation of the heart muscle). Both participants already had long COVID at the time of vaccination.

While the majority reported temporary vaccination reactions, two participants experienced a persistent deterioration in their health and long COVID symptoms after vaccination against COVID-19. *“Since then, I just feel much worse than before” (F2, P2).* Two participants pointed out that they tolerated the vaccination well and did not notice any physical reaction to this intervention.

### Rehabilitation experiences

16 participants stated that they had utilized outpatient (3) or inpatient (13) rehabilitation in the context of long COVID. They received rehabilitation measures with different focuses (including psychosomatic, neurological, and cardiological). Four of them obtained rehabilitative follow-up care in the form of rehabilitation sports. Five participants planned to attend outpatient (1) or inpatient (4) rehabilitation, in which they had high expectations for bettering their complaints. *“I’m going to rehab in four weeks and I’m looking forward to it. That’s when I’m going to leave long COVID and I’m going to come back totally stronger” (I5).* There were large differences between the participants in their experiences with rehabilitation measures. Four individuals reported that rehabilitation helped to improve their health situation and alleviate their long COVID complaints. Two participants perceived the combination of inpatient rehabilitation at the seaside and follow-up rehabilitation sports as helpful. One interviewee had a positive experience with neurological rehabilitation, through which she was able to expand her disease-specific skills. *“I then followed up with neurological rehab, which has helped me a bit in my understanding.” (I20)*.

At the same time, four participants experienced their inpatient (3) or outpatient rehabilitation (1) as unhelpful or counterproductive for their long COVID complaints. They reported that the therapeutic interventions were inadequate, inconsistent, and/or too focused on individual complaints. The participants discussed the lack of focus of rehabilitation clinics on long COVID and the lack of experience of professionals in treating patients with this condition as possible reasons for this.

Two participants pointed out that they had utilized inpatient psychosomatic rehabilitation, in which the topic of long COVID was not considered. *“For me, psychosomatic was the best fit, but as I said, COVID, was not an issue there, was not mentioned at all”* (F1, P1).

## Discussion

### Main results perception of COVID-19 vaccination and vaccination reactions

The 48 participants in this study reported heterogeneous experiences with COVID-19 vaccination. While nine participants were affected by long COVID although vaccinated beforehand, six participants stated that they had not been vaccinated at the time of infection. The number of participants who were convinced of the effectiveness of COVID-19 vaccination in terms of milder COVID-19 sequelae predominated. However, several participants were questioning whether this measure had a health benefit or rather a detrimental effect on them. Of the ten individuals with various vaccination reactions, two reported severe side effects. Three participants experienced symptoms similar to those during the acute phase of COVID-19 (including fever, limb, and muscle pain) and two participants reported cardiac symptoms (myocarditis and extrasystoles) after COVID-19 vaccination. Two individuals perceived a persistent deterioration of their long COVID symptoms as a result of vaccination. These experiences led to a loss of confidence in the safety of COVID-19 vaccines in some participants and uncertainty about how to deal with this preventive service. Two participants also questioned whether the COVID-19 vaccination was causative for their symptoms or, in combination with the COVID-19 disease, led to the long COVID complaints.

### Framing by existing evidence

Several studies indicate that the vaccines against COVID-19 licensed in Europe are highly effective in minimizing severe and lethal cases of disease and are safe to use [52, 53]. In the course of the desired confrontation of the immune system with the vaccine, temporary symptoms such as fever, headache, aching limbs, or swelling at the vaccination site may occur, as reported by some participants in this study [54–56]. Severe side effects or vaccine complications such as myocarditis and pericarditis are very rare [54, 57, 58]. Mentzer and Keller-Stanislawski [58] also point out that such adverse reactions may occur temporally after COVID-19 vaccination but are not always causally related to it. Regarding the long-term consequences of COVID-19 vaccination (e.g., post-vac syndrome) and the effect of this intervention on long-term symptoms after SARS-CoV-2 infection, the scientific evidence is still fragmentary. No clear conclusions can be drawn from the few articles addressing the effects of vaccination on pre-existing long COVID. Several studies suggest that COVID-19 vaccination leads to relief of long COVID symptoms in many affected individuals [59–61]. However, other studies report both positive and negative effects of this intervention [62] or point out that most participants with long COVID do not show symptomatic changes attributable to vaccination [63, 64]. The current data and the uncertainty of some participants in this study illustrate the need for large-scale studies on the effects of COVID-19 vaccination and different vaccines on the symptomatology of long COVID [60]. Consistently, several studies indicate the preventive efficacy of full COVID-19 vaccination in reducing the risk of long COVID following breakthrough infection [26, 62, 63, 65].

To increase trust in the vaccination campaign and public health authorities, there is a need for transparent, timely, accessible, and understandable communication of information on various aspects related to COVID-19 vaccines, such as efficacy, safety, risks, and scientific uncertainties [66–68]. German health authorities such as the Paul Ehrlich Institute have used various tools such as safety reports or red letters to implement active risk communication [67]. It should be investigated to what extent these communication measures reach and are perceived as adequate by the medical staff, the population, and especially population groups with specific safety needs such as people with long COVID.

In a survey of people with immunosuppression, similar to this study, respondents who reported considerable vaccine reactions or side effects after COVID-19 vaccination were less confident in the safety of vaccines. However, the majority of participants in the quantitative study viewed vaccinations positively and had high confidence in vaccine safety. Concerns about vaccine reactions or side effects were also expressed by only a small proportion of participants [69]. Several studies show that, in addition to personal experience with vaccine reactions or side effects, factors such as social and moral norms, lack of trust in government actions, conspiracy beliefs, and rapid vaccine development can lead to concerns and uncertainty about COVID-19 vaccinations [70–74]. Such concerns and uncertainties have a high potential to compromise the effectiveness of immunization programs [75], which in turn would have considerable public health consequences. Therefore, future vaccination studies and programs should take greater account of the attitudes, experiences, and concerns about vaccination of different population groups, specifically of people who are particularly affected by the health consequences of COVID-19.

### Key findings rehabilitation experiences

The rehabilitation experiences of the study participants also showed an extremely heterogeneous picture. Four participants perceived an alleviation of their long COVID complaints following inpatient or outpatient rehabilitation. Two of them experienced the combination of inpatient rehabilitation at the seaside and follow-up rehabilitation sports as helpful. Four participants perceived their rehabilitation as unhelpful or counterproductive for their complaints. As reasons, they cited inadequate and inconsistent therapeutic interventions and, as possible causes, a lack of competence and a lack of specialization in rehabilitation facilities regarding long COVID. For two individuals, no attention was paid to the topic of long COVID in rehabilitation. The 16 participants with rehabilitation experience in the context of long COVID utilized psychosomatic, neurological, or cardiological rehabilitation programs. They did not receive rehabilitation tailored to long COVID in a specialized clinic [76].

### Comparison with other studies

Various publications confirm the perceptions of some participants in this study and suggest that rehabilitation programs tailored to individual needs and abilities lead to improvements in physical symptoms, performance, well-being, and quality of life for patients with long COVID [14, 77–80]. Several studies report that multidisciplinary rehabilitation consisting of physical activity, disease-specific knowledge building, and psychotherapy sessions is helpful for affected individuals [80–82]. Harenwall et al. [82] describe lifestyle management programs implemented by experienced health professionals to optimize sleep and dietary habits and develop personalized self-, energy-, and stress-management strategies as promising approaches for rehabilitative care in long COVID. However, various articles point to the lack of studies with larger cohorts of patients for long-term follow-up of the effectiveness of such rehabilitation measures in the context of long COVID [20, 78, 80]. According to Swarnaker and Yadav [21], observational studies should also be conducted to identify post-COVID rehabilitation needs and multidisciplinary post-COVID rehabilitation clinics should be established.

In this study, the term long COVID, established by affected individuals, was used and all people who had persistent or new onset symptoms more than four weeks after SARS CoV-2 infection were considered together. Accordingly, no distinction was made between people with “subacute/ ongoing COVID-19” (symptoms persisting more than four weeks or new onset [83]) and people with “post-COVID condition” (symptoms persisting more than three months or new onset [84]). In future studies, a differentiation should be made at this point, as it can be assumed that COVID-19 vaccination, rehabilitation measures, and the impact of these healthcare services on one’s health are perceived differently depending on the duration of symptoms.

### Strengths and limitations

This study refers to a sample of 25 adults (one-on-one interviews) and a sample of 23 adults from four focus groups. Recruitment of the two samples was limited to a two-month period in 2022 in each case. In determining the sample size, we followed the literature on good qualitative research practice, which recommends conducting at least twelve interviews [34–36] or three to five focus groups, each with five to eight people [37–40]. Most of the participants are employed persons of German origin with a residence in the federal state of Lower Saxony. Various population groups (e.g., people with a migration history, older adults, or people with a low level of education) are underrepresented in the samples. This study does not represent severe cases of illness, as we excluded participants who could not follow a one-hour conversation from the outset for pragmatic reasons. As we offered both videoconferencing and face-to-face interviews, we were able to increase reachability to participants with barriers such as limited mobility or lack of videoconferencing equipment. The triangulation approach allowed us to take advantage of the benefits of two data collection methods. Longer individual speaking time in the one-on-one interviews provided in-depth insights into the participants’ attitudes and experiences with COVID-19 vaccination and rehabilitation. The group dynamic effects and higher performance of the focus groups [37] facilitated participants’ identification of potential causes of perceived problems regarding these care topics.

## Conclusions

The study participants had very different experiences with COVID-19 vaccination and outpatient or inpatient rehabilitation. Willingness to be vaccinated and participate in rehabilitation was high in these samples. Some participants reported considerable vaccination reactions, and several individuals experienced a persistent deterioration in their long COVID symptoms after vaccination. This led to uncertainty regarding the safety, individual benefit, and/or handling of COVID-19 vaccination. The same number of participants felt that the rehabilitation services they received were helpful or not helpful for their long COVID complaints. Based on the experiences of the study participants, the following implications can be derived: (1) researchers should explore the interaction of COVID-19 vaccination and prior COVID-19, as well as the effects of vaccination on pre-existing long COVID symptoms, as part of controlled trials; (2) policymakers should be more responsive to the perspectives of people with long COVID regarding COVID-19 vaccination in future vaccination campaigns; (3) care planners should build specialized rehabilitation facilities such as multidisciplinary long COVID rehabilitation clinics; (4) care providers should make efforts to increase the skills of their rehabilitation professionals regarding long COVID and develop rehabilitation programs tailored to different clinical pictures.

### Electronic supplementary material

Below is the link to the electronic supplementary material.

Supplementary Material 1

## Data Availability

The transcripts generated during this study are not publicly available as they may compromise research participant privacy. It is however available from the corresponding author on reasonable request within a data-sharing agreement, and subsequent approval from the responsible research ethic boards.

## Abbreviations

COREX: COnsolidated criteria for REporting qualitative research
DEFEAT: DEFEnse Against COVID-19 STudy
MHH: Hannover Medical School
NICE: National Institute for Health and Care Excellence
RKI: Robert Koch-Institute
SARS-CoV-2: Severe Acute Respiratory Syndrome CoronaVirus type 2
UMG: University Medical Center Göttingen
