# Supplementary material for: How do people with long COVID utilize COVID-19 vaccination and rehabilitation services and what are their experiences with these services? results of a qualitative study with 48 participants from Germany
Source: BMC Public Health. 2024 Mar 28;24:915. doi: 10.1186/s12889-024-18380-6 (PMC10976759; doi:10.1186/s12889-024-18380-6)
Supplement: Supplementary file 1 — Supplementary Material 1 [file 12889_2024_18380_MOESM1_ESM.docx]

Supplementary file

**Interview guideline**

Version III (February 18^th^, 2022)

**Demographic data**

Sex:

Nationality:

Age:

Occupation:

Marital status:

Children yes/no; Number (if "yes"):

**A Health situation**

1. Do you remember when you first became aware that you had an ongoing limitation after your corona infection? What were your thoughts and feelings regarding this?
2. What health limitations do you currently have?
3. Do you have the impression that people around you are considerate of your current health limitations and if so, what do you attribute this to?
4. Have you received a corona vaccination despite your infection with the coronavirus?

**B Private / social environment and everyday experiences**

1. Describe your situation at home. What is your everyday life like in your family/partnership?
2. How do your health limitations currently affect your interactions with family, friends, and your partner?
3. Are there people in need of care in your environment whom you take care of?
4. Are you active in the church and what significance does it have for you in your everyday life?
5. What are your hobbies and how do you manage to implement them in your everyday life?
6. Do you do sports regularly?
7. During the corona pandemic, there were government restrictions such as contact restrictions, distance regulations, and masking requirements. How did you experience these stipulations?
8. During the corona pandemic, some people strictly followed the corona rules, while others showed more of a negative attitude and questioned the extent of the corona crisis. What are your thoughts on this? Has your opinion or behavior changed after you infected yourself with the coronavirus?

**C Occupational context**

Please tell me about your current occupation and the collegial environment in which you work.

1. Has your occupational situation changed due to health limitations following the corona infection? If so, what is different or new?
2. How did you experience the period of the corona pandemic in your occupational environment?
3. Do you have the possibility to implement home office and how did you manage this during the corona pandemic?
4. How did you experience your supervisors in dealing with the corona pandemic?
5. How do you manage to combine your current health limitation with job requirements?

**D Perception of healthcare in Germany / corona pandemic**

1. How do you experience medical support or advice regarding your current health limitation?
2. Do you know other people who have been infected with the coronavirus and have similar symptoms as you? Do you have any contact with these people?
3. How do you currently feel about the federal and state governments in terms of dealing with the corona pandemic?
4. What has been your experience regarding education on vaccination for corona protection?

**E Life perspective**

1. The corona crisis has been going on for over two years now. What has changed in your circle of friends during this time? What has remained the same?
2. Do you feel that you can currently do the things that are important and meaningful to you?
3. What strategy have you found for yourself to deal with your current health limitation?
4. Looking back at the time before you were infected with the coronavirus, would you behave differently looking back at your health limitations today?

**Moderation guideline focus groups**

1st stimulus with question (recorded radio report on the topic of COVID long-term consequences in everyday life and medical support in post-COVID (up to minute 02:40 [37])).

**What thoughts do you associate with what you just heard in the report?**

2nd stimulus with question.

**When you think of health limitations such as concentration problems and fatigue - what impact do they have on your working life, your family life, and your social contacts?**

3rd stimulus with question.

Statement of an interviewee: "I have to accept it somewhere, but at the moment I feel left alone by the doctors from the point of view that just nothing happens except that I am put on sick leave. And that's a bit like sitting here at home and doing nothing [...]. I say yes, it's good that at least with the doctors, not all of them, but with the doctors where I've been so far, it was mostly accepted and taken seriously, but that they dealt with the issue or said 'Come on, we'll do it now. We'll look at something else now, we'll try something or other.' I didn't experience that, not at all. No interest at all in helping in any way."

**What do you think about this?**

4th question.

**Imagine you would live in a world with unlimited resources: What would perfect care and support for people with your health limitations look like there?**

**Optional questions:**

1. When did you become infected with coronavirus?
2. Did you receive a corona vaccination and if so, when was it?
3. How do people in your environment take your health limitations into consideration?
4. How do you evaluate the political measures to protect against the Corona pandemic? In what ways do these measures have an impact on your situation in the context of long COVID or post-COVID?
5. What strategies have you found for yourself to cope with your disease and to what extent did you develop strategies to compensate for limitations?
